# Interacting institutional logics in general dental practice^☆^

**DOI:** 10.1016/j.socscimed.2013.05.038

**Published:** 2013-10

**Authors:** Rebecca Harris, Robin Holt

**Affiliations:** a The University of Liverpool Department of Health Services Research, UK; b University of Liverpool School of Management, UK

**Keywords:** England, Institutional logics, Dental practice, Professionalism, Commercialism, Institutional theory, Autonomy, Accountability

## Abstract

We investigate the organisational field of general dental practice and how agents change or maintain the institution of values associated with the everyday work of health care provision. Our dataset comprise archival literature and policy documents, interview data from field level actors, as well as service delivery level interview data and secondary data gathered (2011–12) from 16 English dental practices. Our analysis provides a typology of institutional logics (prevailing systems of value) experienced in the field of dental practice. Confirming current literature, we find two logics dominate how care is assessed: business-like health care and medical professionalism. We advance the literature by finding the business-like health care logic further distinguished by values of commercialism on the one hand and those of accountability and procedural diligence on the other. The logic of professionalism we also find is further distinguished into a commitment to clinical expertise and independence in delivering patient care on the one hand, and concerns for the autonomy and sustainability of a business enterprise on the other.

## Highlights

• A case study of English dental practice explores how multiple competing logics are managed.
• Practice ownership responsibility is identified as a distinct logic.
• Dental practitioners were found to readily move between four different logics sometimes invoking several at the same time.
• Commercialism logic was less in conflict with professionalism logic than with population health managerialism.

## Introduction

Market-based health care reforms, emphasising reductions in state involvement, creating incentives for greater efficiency through competition, and moving from ‘public service ethic’ towards private management styles, have been sources of concern in the UK and beyond (Segall, 2000). There is pervasive unease that demands for greater care efficiencies traduce professional standards; logics of cost can restrict ability to give the best available care, and logics of commodification belie the idea of a patient being special, unique even (Gabriel, 2009). Relman (2007) for example, writes ‘the essence of medicine is so different from that of ordinary business that they are inherently at odds’ (p.2669), predicting medical professionalism cannot survive a commercialised health care market. UK general dental practice is, however, somewhat distinct; whilst being part of the National Health Service (NHS), provision has been governed using quasi-market principles for many years and a mixed economy of publicly subsidized and fully out-of-pocket paid (private) care exists (Taylor-Gooby, Sylvester, Calnan & Manley, 2000). Our study investigates the institutionalization of this joining of professional and commercial ‘logics’, specifically recent developments, from the organizational perspective of providers, the dental practice.

At the outset we approach our study as one concerning the institutional work of agents in which it is neither individual agents nor institutional structures, but their mutual expression, that forms, sustains and upsets the logics by which everyday activity finds legitimation. Thus we investigate agents absorbing, adapting or challenging prevailing and emerging institutional expectations surrounding innovation, accountability, economic efficiencies, well-being and professionalism. One such logic can dominate (Greenwood and Hinings, 1993), for example the prevailing pre-eminence of clinicians in health care decision-making (Battilana, 2009), which then gives way, or is accompanied by ‘rival’ logics, associated with commercialism say (Currie, Lockett, et al., 2012, Reay and Hinings, 2009).

In the context of UK dental practice we too find multiple logics associated with clinical professionalism and commerce. The professionalism associated with clinical expertise we find extended to a concern with preserving the viability of an enterprise (the practice) upon whose flourishing the livelihood of employees and the integrity of the wider local community depend. The logic of business is also refined, as through making sense of institutional pressures to be a business, dental practices experience values associated with both accounting probity and commercial innovation. In some instances we find dental practice accommodating all four forms of logic, readily moving between them, or invoking them at one and the same time.

Our paper proceeds as follows. We introduce institutional work theory and its use within the field of health care, into which we also bring other studies of dental practice touching on questions of institutional reform and evaluation of care. We then describe our secondary and interview data. Our findings we organize into a typology of logics and discuss their implication for understanding how evaluations of health care provision in dental practice, and more broadly, are configured through mutual expressions of structure and agency.

### The institutional setting of UK dental practice

Almost 80% of the 31,000 practising dentists (40% are female) in the UK work in dental practice (Kravitz & Treasure, 2009). Since the establishment of the General Dental Service (GDS), practitioners have acted as independent contractors to the NHS. They own their own premises, employ their own staff and pay expenses (like materials) from income. Under NHS contractual terms practices are free to provide as much or as little NHS care and private care as they wish. The vast majority of practitioners do at least some NHS work; on average NHS practitioners spend 75% of their time on NHS work. Whilst the majority of UK practitioners work alongside other dentists in professional partnerships (P^2^), (Greenwood, Hinings & Brown, 1990), a third of the 11,000 practices are solo practices, where just one dentist owns the practice and provides care (Kravitz & Treasure, 2009). Government removal of restriction on the number of Bodies Corporate (DBCs) in 2006 made market entry easier for practices owned by external commercial organisations, giving rise recently to several large chains of DBCs, trading on stock markets and owning upwards of 300 practices. Supplementary material.

The GDS is one of the few areas of the NHS where patients are involved in co-payment, meaning commercial and health-care concerns are intimate. Legislation enacted in 1951 allowing patient charges for dentures became the first charges of any kind to be levied for NHS care (King, 1998). This was quickly extended to allow for patient charges for other types of treatments. This precedent of co-payment has been a feature of NHS GDS care ever since.

Studying the established and emerging criteria by which dental practice is evaluated involves concern for multiple agents (clinicians, managers, suppliers, patients, politicians, commissioning bodies, professional bodies etc.); institutional settings (public policy agenda, health and safety procedures, market forces, etc.) and norms (professionalism, affordability). There is no dominant agent or institutional force, rather agency is experienced in following established institutional settings, and institutions are animated, deepened and resisted in being taken-up within ordinary lives. In UK dentistry this has evolved into a mix of publicly/privately funded provision. Following DiMaggio and Powell (1983), we can identify such institutional settings as an organizational ‘field’ governed by prevailing logics, often tacitly expressed, that are beyond the gift of individuals to change, and which govern what effective care means. A field acts as a ‘common meaning system, where participants interact more frequently and fatefully with each other than with actors outside the field’ (Meyer, 2010, Scott, 2001: pp. 138–139). Thornton & Ocasio (1999), p. 804, for example, define institutional logics as ‘the socially constructed, historical patterns of material practices, assumptions, values, beliefs and rules by which individuals produce and reproduce their material subsistence, organise time and space, and provide meaning to their social reality’. Logics are the predominating beliefs that create connections and a common purpose allowing those within a field a sense of grounding, orthodoxy and habituated normalcy; it is through logics that organization, actors and agency are woven into one another (Friedland & Alford, 1991).

To study health care logics is to investigate how agency, whether from individuals or organizations, commits, adapts and challenges prevailing structures of symbolic value and evaluation (the criteria by which care provision is considered effective) from within the field and beyond, whilst accepting agency persists only by being institutionalised within such structures (Lawrence et al., 2009, Lawrence et al., 2011, pp. 1–28; Meyer, 2010). By agency is meant the capacity to effect somehow the social world; professions, for example, being institutional agents who shape, legitimate and distribute the knowledge and practices governing activity like health care provision (Scott, 2008). This institutional perspective fosters analysis of the ways in which agents enact their environment and are similarly acted upon by the same environment, in everyday work settings (Lawrence et al., 2009, pp. 1–28).

So understanding how health care in UK dental practice is appreciated we attend to, and look beyond, specific decisional responses to immediate problems of co-ordination and control; accepting that institutional structures like professional ethics, profit-based market forces and prevailing ideas of ‘health’ have meaning outside of any specific individual's interpretation. Institutionalized conditions form the non-negotiable grounding allowing agency to occur. These processes of institution cannot be reduced to the instrumental logic of a decision, to institutionalize is to infuse the field with values that pertain beyond the immediate technical requirements of tasks at hand (Berger & Luckmann, 1967). For example, a patient's decision to open their mouth whilst lying prostrate in a chair is only possible in a setting of habituated expectations concerning: the competence and integrity of professionals; the desirability of healthy teeth; the probity of payment mechanisms, and so on. Yet none of this institutional settlement is immutable. No sooner is such a field posited than its dynamic nature becomes apparent (Lawrence et al., 2011). Fields are permeable, influenced by logics from other fields (e.g. employment law and litigation systems in legal fields) and from within as actors espouse multiple logics (e.g. personal dogmas). In being enacted, disruption can occur as actors take the logics on, tarry with them, innovate even. Such enactment is often open, with agency effects being more nuanced than simply resistance to or acceptance of institutional values (Currie, Lockett, et al., 2012).

Several logics may co-exist within an organisational field, although one is generally dominant. Kitchener and Mertz (2010), for example, found a dominant logic in US dental practice of clinical excellence coupled to a top-down, well-structured governance systems whereby each dental practice was led by the dentist (typically male) with other actors' fitting into allotted roles. Competing with this, though, was an emerging logic associated with the agency of hygienists, who, seeking alliances within and beyond the organisational field (for example with consumer groups representing the disadvantaged, and with public health professionals) wanted to extend provision into disenfranchised areas and to constitute alternative practices askance from the traditional professional logic associated with dentist-governed care.

Such struggles are experienced as new practices and norms - perhaps prompted by breakdown events, new actors, shifts in leadership, or new technologies - are advocated, and established ones defended, or amended (Kitchener, 2000, Meyer, 1982). How actors respond to institutional pressures varies, and in this process of struggle and resolution, actors are understood to gain skills and capital for future institutional involvement (Oliver, 1991, Reay and Hinnings, 2005). In the course of such, the meaning and priority of activities can change given differing logics, with some becoming redundant or anachronistic, and others lying dormant, to be resurrected at a later time, and others surfacing. Reay and Hinings (2009), for example, suggest distinct logics in a field can play out competitively as actors seek to champion and assert one set of values over others as in the case of Kitchener and Mertz's study, or more broadly by Relman (2007), subversively (as one logic works ‘under the radar’ of an espoused, dominant logic), or collaboratively (as adherents find structural stability in a form of balanced ‘truce’ despite varied and sometimes conflicting understandings). Reay and Hining's (2009) study examines a case of collaboration, though here in the more general organizational field health care provision in Alberta, Canada, finding uneasy but functional truces between logics of professionalism (embodied in values of expert independence, patient care priority and clinically-led provision) and business (embodied in values of customer satisfaction and cost effectiveness). ‘Business-like health care’ concerns the legitimacy of services delivered at lowest cost, based around ‘consumer’ relationships between the patient and health care provider; the overall goal being to provide efficient and effective services. The emphasis here is on delivering care with the population set adrift quasi-market forces in mind, rather than individual patients. They contrast this with medical professionalism, where the profession is responsible for controlling the quality of service provision as well as certifying new approaches when they become available, and the individual clinician occupies a position of authority vis a vis the patient and service management. Central to professionalism logic is the dominance of (protected) clinician–patient relationships.

The tension between managerialism and professionalism has been described in studies describing conflicting institutional logics in health care (Currie and Guah, 2007, Kitchener, 2000), with business-focused institutional logics resisted by professionals delivering front-line services. Other authors, however, find medical professionalism logics more nuanced. Hanlon (1998) identifies separate logics associated with professionalism. He distinguishes ‘individualistic professionalism’ (the provider is a ‘gentleman’, careful of his word and reputation, providing a service to people who can pay); ‘social service professionalism’ (democratized professionalism providing services for everyone); and ‘commercialised professionalism’ (stressing the value of managerial and entrepreneurial skills in providing a cost-effective, quality service), all of which might persist in evolving mutual influence rather than the obliteration of one by others (Cooper, Hinings, Greenwood & Brown, 1996). Commercialism can also be nuanced, with some arguing care is a commodity whose price is governed by forces of scarcity and felt want and patients become active choosers rather than passive recipients (Ozar, 1984). What, then, are the prevailing commercial and clinical logics being experienced in UK dental practice?

## Method

We conceptualised dental practice as an organisational field consisting of providers (dental practices), resource and product consumers (patients/customers), regulatory agencies (commissioners, government and professional organisations). Actors within this field (those interacting with each other frequently and fatefully (Scott, 2001) include: government agencies and departments, professional bodies such as British Dental Association (BDA), dentists and other dental team members; dental accountants and legal representatives; commissioning bodies and patients. In line with existing institutional studies of health care practice, at the outset we conceptualised the prevailing logics as being broadly grounded in ideas of effective patient care.

National research ethics approval (Reference number 10/H1011/38) and NHS research governance approvals were obtained for the study. Our dataset comprised archival documents, as well as interview data from actors both at the service delivery level (e.g. patients, commissioners, dental practitioners) as well as field level actors (government, professional organisation representatives at the local and national level, dental corporate providers at Chief Executive level). The archival dataset comprised official documents and news releases published by the government and professional bodies between 1980 and 2012. These were retrieved by hand and online searches of government and professional publications. Their content allowed us to prompt discussion in interviews with field level actors (*n* = 19). These interviews were semi-structured, grounded in concern for understanding the interviewees' sense and experience of delivering ‘effective care’, from which we were able to identify how dental practices enacted prevailing logics associated with clinical excellence and sound business practice. Over 2011–12 we collected service level data from 16 dental practices in northern England covered by 6 Primary Care Trusts (PCTs, currently the organizational form responsible for managing contractual relationships associated with NHS provision). Data included transcripts of audio-taped interviews with dental practitioners (*n* = 17), dental team members (*n* = 11), patients (*n* = 39), commissioners (*n* = 15). Six dentists interviewed provided mainly private care and 11 dentists provided both NHS and private care to varying degrees. Dental practitioners and commissioners were interviewed periodically to follow key events over the study period, such as contract reviews. Non-participant observation data were also gathered of meetings between practitioners and commissioners and dental practitioner network meetings. Documentary evidence relevant to case study events was also gathered, such as e-mail correspondence, minutes of meetings etc; photographs of practices (internal and external, for example including signage). Where interviewees spoke of the dental practices being in conflict with commissioners during the study period, these were studied with the greatest intensity. In total 8 practitioners and 4 commissioners were interviewed more than once. In all 57 interviews were conducted at service delivery level. Using NVIVO 9.2, transcripts were initially open coded, then re-coded according to theoretical concepts and emergent categories. Transcripts were analysed to code data expressing values, norms, beliefs, interactions and behaviour of actors broadly in relation to concerns with care, accountability and commerciality. Analysis followed a ‘constant comparison’ method, where provisional themes emerging from one set of data were subsequently compared with other transcripts. The process of analysis was iterative, with emerging themes discussed with a wider analysis team alongside further reading of the literature, leading to refinement of codes and categories (Numagami, 1998). We have coded data extracts in the results as follows: the first letter denotes separate PCTs; second letter is D if the participant is a dentist, C if a Commissioner and P if a patient. The third digit denotes number of participant. PCR represents an interview held as debrief following contract review meeting between practitioners and commissioners.

## Service-level data: logics in the dental practice field

Analysis of the archive data finds the field governed by three, not two, related logics: Medical professionalism (care through technical expertise and ethical standards); Business-like health care (efficiency, effectiveness and transparency); and Commercial logic (using terms like customers not patient, or describing care as a commodity). Table 1

**Table 1:** Indicative statements relating to institutional logics from archival data.

Medical professionalism logic	Business-like health care logic	Commercialism logic
*Providing dental care as a dental professional*	*Delivering dental care according to principles set by public funders*	*Supplying dental care as a commodity*
**Dentists:** Do we wish to be considered as entrepreneurs in the market place – and treated accordingly? Or as a learned society serving society and accordingly looked after and rewarded by society? (Welsh Valley practitioner, 1985) **Dentists**: If one relies on patients who have ‘shopped around’, despite standard NHS fees, then one must expect patients who are more interested in ‘cheap’ treatment. In the words of a gentleman who called here recently ‘I would not want to go to a dentist who had to advertise’ (Crocker, 1986) **Dentists:** It is an indisputable fact that professional standards are absolute and therefore not negotiable. Standards are limited only by the corpus of knowledge and achievement of the age (Bosley, 1987)	**Dentists:** We applaud both equality and excellence, but we are not at all sure if we can have them both (Sear, 1984) **Govt:** The available money buys for patients, both a decent level of quantity and decent quality of care and treatment, while providing dentists with a reasonable return for their efforts (Bloomfield, 1992) **Govt:** Money spent on dentistry should be used effectively and efficiently to improve oral health. Need rather than demand should be the focus of the system. (Department of Health, 1994) **Govt:** PCTs should commission such services as are necessary to secure access to a high quality NHS dental service and to improve oral health and address inequalities (Department of Health, 2002).	**Dentists:**. We are going to have to market our services and persuade our patients to part with their discretionary dollars. Many say that this debases the profession – *hoosh*! (Eilertsen, 1986) **Dentists:** An increasing quantity of dentistry done in the UK has little to do with general health. … Why cannot dentistry be considered just one more consumer good? (Leader, The Probe, 1996) **Dentists:** No one enjoys going to the dentist and patients do not buy dentistry [oral health] as a commodity. They buy an attractive smile and the benefits of oral health in that order. ‘Need’ hangs around the profession's neck like an albatross (Gordon, 1997) **Dentists:** To give up your freewill and control of your business to bureaucrats and politicians would be a gross dereliction of duty to yourselves, your staff and most of your patients. (Rowe, 2006)

To gain richer appreciation of how these were currently experienced, and further refine them, we used analysis of service delivery level data. Going from these data back to the logics conceptualized using analysis of archive data, and back again to interviews (Orton, 1997), we then found four distinct yet inter-relating logics we conceptualized as: ownership responsibilities, professionalism; population health managerialism; entrepreneurial commercialism. Table 2

**Table 2:** Multiple logics in the dental practice field developed from service level data.

Ownership responsibility	Professionalism	Population health managerialism	Entrepreneurial commercialism
**Composite principles**			
*Authority and managerial responsibility for practice staff, reputation and servicing patients and community*	*Clinical excellence, altruism, best interest of patients, patient advocacy, technical knowledge, professional responsibility and self-governance*	*Trace and explain public expenditure; govern good practice.*	*Trading and opportunity with a focus on sustaining and developing a profitable business*

**Expression**			
Try to sustain the business long term for the sake of staff and patients	Patients are treated according to technical and ethical values	Patients are treated as a unit	Patients as a source of income
		Feel part of the NHS	
Close relationships with staff. Sub-contracting is risky	Give the patients authoritative options	Be accountable via hierarchical bureaucracy for what you’ve done and why	Conscious of the market and consumers' wishes.
Ownership is beyond owning the enterprise - it concerns setting practice ethos	Gatekeeper – police what is available on the NHS	Meet targets	Excite wider demand
		Resources governed by need not demand	
Concentrate on reputation of the practice amongst the local community	Treat NHS and private patients the same	See remuneration not based on balancing income and expenditure for individual patients	Commercial GDPs view income as ‘swings and roundabouts’
Close relationships with patients ‘coal face’ built on family/friends Emphasis on ‘our’/’my’ patients	Assert patients' best interests, charge structures second	Strategic priority (public policy) orientated	Range includes piece rate GDPs
		Population prevention strategies	
	Keep abreast technically	Sub contract to others	Conscious of branding
	Individual clinician's are responsible for deciding what is best for the patient	Dispassionate, issue-based decision making	Business entrepreneurship
	Do not criticise other clinicians		

### Ownership responsibilities

Dental practices are often considered independent enterprises whose viability is the responsibility of the partners. There is a palpable sense of the practice providing a livelihood for employees, and of its providing an important community service. It is separate from values, beliefs and rules related to clinical autonomy insofar as the expertise is managerial, close to leadership, and the sense of duty is better described as arising from ownership of a practice for which they alone are, autonomously, responsible. Values concerning ownership define ‘the way of doing things’, or the ‘ethos’ of the practice, ranging from fostering a sense of collectivity amongst staff in often stressful environments to tending the physical assets of the practice, as well as building wider community links. Such autonomy is also felt negatively, for example through suspicion of employing ‘strangers’ or sub-contracting work. This is risky because the dental practice is a close-knit, supportive community. Problems with dental team members place demands on the managerial skills of dentists. There is therefore recourse to long-standing employees and family members. In these ways, the enterprise carries a sense of distinction extending beyond its capacity to generate rents.

We are an independent contractor but I'm not removed from the practice, I'm not a corporate body where I'm sitting in an office, who has several dental practices but [I'm] working “on the coal face” as they say. (BD3, fully NHS)

In dentistry personal relationships are extremely strong. (CC2)

I'm a little frightened of employing the younger generation, you know as two of them were terrible. And I'm not very good at managing the unruly who would need a lot of time to put right. (DD2, fully NHS)

I think one thing here is we are a small community. You've got to remember that. That the people we see in the waiting room – a percentage of them are friends. You see them in the shops. (CD3, 90% NHS)

We try to care for them and there are a lot of incentives to do that because they are not only patients but they are part of the community. Of course anyone would want to look after them in that position. (CD3, 90% NHS)

Same practice manager, the same partners but the partners were always silent because they felt they had to support [the former principal]. But they have a completely different relationship now with the PCT. (DC1)

He just wants to be left alone and he thinks if he reaches his UDA target everything's fine, never mind all the other issues that go with it. He just wants to be left alone. He's the dentist, his wife is the receptionist/ dental nurse - end of story. (DC1)

We were at a meeting once and the guy, the [PCT] financial director said “We want our practices to do it this way” and I said “What do you mean, your practices? It's our practice – you haven't put a penny into this practice. We sub contract and do treatments and you pay us. We bought the building, we put all the equipment in, we pay all the staff, how come it's your practice?” (DD1, 100% private)

I don't like the idea of being told what I can or can't do by a third party… “Use this software” or “You don't do this”. Even if it's nothing to do with your clinical practice – I don't like the idea of that. (ED1, 20% NHS)

### Professionalism

This long standing and well-attested logic espouses dentists, as experts, always acting in the patients' best interest, and according to professionally defined standards of care as judged by the individual clinician at the dental chair-side. The belief that each clinician is individually responsible for the care that they provide according to this threshold means daily clinical practice of other practitioners is rarely challenged. The reluctance reveals the skills gained through apprenticeship into professionalism are neither gained nor lost lightly.

I try and keep the ethos which is straight forward and simple. I'm not really bothered too much clinically if the patient can pay for treatment or not. I'm interested in whether or not I can look after their mouth. (AD1, fully NHS)

The professionalism we adopt is of the highest standard because we're using our skills to teach a junior colleague to make sure that they have not only the quality of professionalism but the capability to be self determining at the end of the one year training. (AD1, training practice).

To me it's all… I look at it and to be honest sometimes I think I don't really know what that percentage means and I don't understand it. But to me I am not bothered about things like that. I mean, I look at it and our patients are 100% satisfied with what treatment they are getting. (AD2, 20% NHS)

I personally don't like to sell too much. I like to treat the patients – do the care that's needed to them and know that we are doing it on the NHS and avoid too much of the selling aspects because then it becomes like… you know.. you have to sell as well what you are providing. (BD3)

My values stay the same (for private or NHS patients). It's a professional part of being ethical. My values don't really change. If I started making decisions based purely on economics (for example) then I'd start to question my ethics. (CD1, 20% NHS)

I'd be at a loss to justify it, there was no justification. I've got my patients best interest as my best interest. (CD1, 20% NHS)

I am not in a position to comment on that because we look after our own patients here, I don't inspect other practices. (CD3, 90% NHS).

My customer is the person who wants my services, that is my patient - NHS or private, I don't differentiate the way I treat people. (ED1, 20% NHS)

### Population health managerialism

Value-for-money assessments pervade all modes of NHS care provision, especially peripheral and commercially inured dental practice. For many interviewees value-for-money was synonymous with low cost tendering rather than doing more with less. There was appreciation that against a backdrop of scarce resources achieving equity of provision was important, involving reporting and record-keeping to ensure actions are transparent. This logic, then, is one of being held accountable, but to which constituency? Interviewees' comments gather around achieving NHS targets and being open and transparent to commissioners and other policy makers, and patients. These targets and procedures, however, were considered often inconsistent, requiring tactical abeyance. A basic expression of this comes amongst those acknowledging income generation through NHS provision being an experience of ‘swings and roundabouts’, with less profitable interventions being offset by more highly remunerated ones.

We had a practice who were delivering not even gold standard, but probably platinum standard which was very, very nice but completely unaffordable. (CC1)

We handle it by keeping a close eye on our UDAs and we know when we're coming up to the number and then we'll stop seeing children. (AD2, 20% NHS)

In order to improve the quality of care we had to shed some numbers. (CD1, 20% NHS)

There is a daily count and monthly count. And we'll just know as we go along, and it just seems to work. (CD3, 90% NHS).

It is a business at the end of the day, you do have to ensure you're getting the best costs when it comes to materials but as foremost I still myself as a public [servant]. (BD3, fully NHS)

Well I'm more or less relieved when I've more or less achieved my stupid targets because that really would be the last straw really, if I didn't – which is sad. Well, it's pointless. I mean, I don't know what the point of worrying is (laughs). It's not achieving anything other than trying to stay financially viable. (DD2, fully NHS)

The guy whose private crowns I did yesterday, he could afford the private crowns but he doesn't need the crowns. He's got a bit of gum recession and you can see the margins on his front teeth, his wife is giving him earache and I can't turn around to the Treasury and say “This guy's wife is whinging that he needs 2 crowns, stuff the bill”. On the other hand you could be in a situation where if the PCT is penalising you for underperforming by 4% or more in my UDAs, maybe I could turn a blind eye and do those crowns because I need the 12 UDAs. It's that sort of dilemma, moral dilemma, and that's where you've got to try and be quite professional about it. (BD1, fully NHS)

Well if you're a GDP and in the NHS you know you've got targets to reach, you've got to see so many patients and all that to get your money and it's no wonder that some people (like this practice), sort of decided, well we'll just go private because we're not getting paid enough for what we're doing. (AD2 P4)

### Entrepreneurial commercialism

There is growing awareness of dental care offering commercial opportunity. The practice was often spoken of commercially, a commercial space of service delivery, income and profitability. Some spoke of opportunities to expand (grow practice through acquisition or joint ventures), entering new areas of business (cosmetic services), and investing in marketing (advertising, sponsorship).

I am a businessman and I initially thought of this contract on day one as a business. Trust me I'd be a lot more rapid in terms of marketing but I didn't want to peak the practice because it's essential to be positioned. (AD3, fully NHS)

We have the best opportunity to work one on one with anybody because they're a captive audience. Sometimes it's difficult for them to speak… That's OK, that's great, you can tell them whatever you like, they can't argue. But you can get a point across. You've got someone sitting in a chair one on one for half an hour. You should be able to sell them anything… (BD1, 95% NHS)

We have xxx who was just sitting in the corner there, she is our on-site business manager and she looks at turnover, our expenditure, keeping material costs sensible, finding the best deals on bills, services. (BD2, 100% private)

The public sector, maybe they don't deal with the money side of it as much. I'm not really sure, because I sit and look at profitability of every single site on a monthly basis… whether a public sector servant does that…? (CD2 corporate)

And we didn't want to be branded NHS either. I can remember being at a meeting and I said “Why would you want to be part of a brand that doesn't have a very good reputation?” Boots – yeah (laughter) – not the NHS. It has got a brand problem, the NHS. (DD1, 100% private)

It's not limited in any way. The limit on what we do is what the patient is prepared to pay for as opposed to practical limitations. (DD1, 100% private)

A lot of dentists don't see the wood for the trees, they don't realise how they can provide better dentistry and hey, make a living for themselves. (ED1, 20% NHS)

## Discussion

By identifying the experience of four interrelating logics, two aspects are worth elaborating arise: their interaction and their contingency. Dental practitioners enact and cope with differing logics, not merely in an ‘uneasy truce’ of one bloc set against another (Reay and Hinings, 2009), but are interweaving threads running throughout their everyday activity. This interaction we find quite explicitly in the practice of some dentists (BD3 for example) fully aware of the practice as a business, yet also avowing a status of public servant and resistant to the idea of his selling a service. For all actors, and especially dentists, the organizational field appears marbled with commercial logic; even those eschewing its values acknowledge its defining a collective proper space within which they must act. Indeed, for many, commercialism appears less in conflict with professionalism than does population health managerialism, notably the bureaucratic and inefficient structures detailing forms of apportioning of care, often with little regard for the ordinary experiences of patient need. So (notably BD1 – in the set of quotes relating to population health managerialism) the tension of ‘facing the patient’ and deciding eligibility for NHS treatment brings the experience of pressure from targets given by commissioners into tension with professional values putting care above affordability, as well as commercial and business autonomy values arising from the satisfaction of patients and maintaining patient fidelity. Here all four logics are being woven. Arguments concerning whether commercial logics (Relman, 2007), or business-like health care (Segall, 2000) are incompatible with professionalism are seen as too simplistic in the light of this more subtle representation.

Our data suggests that the cost-conscious logic of delivering value-for-money care espoused through population health managerialism would be better ensured with closer ties to strict commercialism, rather than pursuing with public sector auditing and commissioning. This is in keeping with the argument put forward by Hanlon (1998), that while public and private sectors derive their economic and ideological basis from different sources (namely the state and the market), the two sectors share much in common, the experience is not readily reduced to conceptual opposition. Entrepreneurial commercialism resonates with, as much as it resists, the logics on professionalism and ownership responsibility. Though many actors sustain grounding commitments to professionalism and, by implication, place overtly commercial ideas of care second, they all acknowledge the responsibility for sustaining viable enterprises. The commercial can erode a commitment to provide the best possible care, forcing ability to pay onto the relationship, yet some dentists acknowledge counterbalancing forces, for example an increased sensitivity to patient demand. Their expertise is negotiated in conversation with patients, and the criteria by which care is understood expands. As a result of the pull of commercial logic, dentists understand what is ‘wanted’ by patients in terms of specific services (tooth whitening, straightening, filling colour). Commercialism also feeds into a sense of autonomy, with dentists increasingly aware of patients as the source of income from which their livelihood springs. This relational awareness extends beyond the meeting point at the dental chair, with practices well aware of being sustained by reputation in the community, creating a persisting sense of fidelity between practitioners and their patients outside of the immediacy of surgery exchange. These findings expand on Doolin's (2002) study of hospital clinicians in New Zealand that draws similar conclusions. Whilst the norms and values of enterprise and entrepreneurialism marble discourses of public-sector reform, it is within the private practice of clinicians where professionalism and enterprise are often less in conflict.

Another apparent weaving of logics, though here more antagonistic, was a tension between ownership responsibility and population health managerialism. This is not a new theme in the literature relating to medical practice (Harrison and Dowsell, 2002). Whilst government involvement in health care delivery has been diminishing in the UK in an era where health policy is geared towards an increased use of competition and markets following an claimed effect of reducing costs and maintaining quality; in the USA, reforms have moved towards greater government control, with the passage of the Patient Protection and Affordable Care Act (2010). Models of UK and USA dental practice delivery have mirrored this change, moving the systems closer together (Currie, Pretty, Tickle & Maupome, 2012). In the UK, following financial pressures in the 1990s which diminished public funding for dental care, dentists have increasingly grown the private element of their practice and reduced their NHS commitment. The UK, a system once dominated by public sector provision under central government control is thus becoming more akin to dental practice in the USA. There dental care is delivered via a decentralised system dominated by private enterprise, although there too, there are institutional pressures (sometimes resisted by dental practices) to introduce a public health approach, where prevention of disease is prioritised, a diverse workforce is used, including hygienists, and coverage of the population is increased (Currie, Pretty, et al., 2012). Our study helps understand where tensions lie when population health managerialism, which represents an increase in accountability, encounters strong resistance from professionals concerned about autonomy and ownership responsibility.

The constant interaction between logics also suggests their being contingent, *viz* that certain logics predominate depending on prevailing circumstances. Cooper et al. (1996) describe how, whilst logics provide coherence and meaning in the form of value norms and procedural standards, they are only ever sedimented through structures as processes. New organisational forms arise, differing technologies and hence actors arrive, economies expand or implode, all of which finds actors having to work at weaving the logics by which they work continually. The idea of care is processual, infused with settlements and tensions playing out through history and across different spaces. Structurally, some UK dental practitioners, for example, clearly accepted new roles and identity as businesses whilst others were more reticent. Advancing on Scott (2008), this acceptance is not readily reduced to an either-or opposition. For example, some interviewees spoke of the emergence of new structures such as Dental Bodies Corporates that organise care to allow some less commercially orientated practitioners to continue practicing, outsourcing business pressures by relinquishing their partnerships and enterprise to professional management, and allowing them to concentrate on medical expertise. Equally, moves to independent private practice provided a route for more commercially minded practitioners to avoid the pressures of population health managerialism, whilst still maintaining allegiance to the logic of ownership responsibility whereby care is appreciated as community duty and extends beyond the medical to include contributions to the local economy. This sense of connection extends and expands the experience of providing care on a day-to-day basis, here the logic changes the practice, and changes with it. So whilst a logic of professionalism finds clinical expertise paramount and unimpeachable, many dentists were discussing care as something that was emerging from experiences of engagement rather than retaining clinical distance; expert views were no longer being imposed from patrician distance. Following Boiko, Robinson, Ward and Gibson (2011), and nuancing the conclusion of Battilina (2006) concerning the continued dominance of clinical perspectives on the idea of care, we found dental practice being configured by reflexivity in care. Among many practices the feelings of patients - their experience of problems and solutions - carry weight; it is not simply the dental ‘look’ that counts. So talking through options with patients, discussing side effects, showing care as an array of possibility (with differing price implications), and in general being aware of care from the patient's perspective, were very apparent. The idea of there being a separable source of controlling agency within the organizational field became a very distant one.

In summary, in addition to adding a more nuanced typology of logics to the field of dental care, our study also advances on others by suggesting two modes by which these logics are experienced: constant interaction and contingency. In this way our study contributes significantly to overcoming the hegemony/resistance categorisation of logics that has hitherto characterized the field of studies investigating the impacts of managerialist logics upon professionalism in health care (Numerato, Salvatore & Fattore, 2011). By following an institutional work perspective, and investigating multiple agents (both within and across different forms of organization) we work beyond those macro theories ascribing unitary forms to the prevailing logics by which different actors appreciate and assess the idea of care. Following Numerato et al. (2011), we have found it is not so simple as placing the actions and experience of clinicians and managers on continuum from resistance at one end to compliance at the other.

## Conclusion

Our study develops the work of Reay and Hinnings, 2005, Reay and Hinings, 2009 and follows their call for studies in different organisational fields where multiple logics exist, particularly where actors hold strong identities and sources of power that facilitate their ongoing independence In this we follow Battilana's (2006) call to expand the level of analysis to broach organizational and individual levels, touching even those of community pressure. We also add to Kitchener and Mertz (2010) in creating an institutional study of dental practice, though here in England, and with an emphasis on the institutional logics governing the provision of health care. We assume that well attested logics associated with clinical professionalism on the one hand, and business on the other, are sufficient to convey the experience of dental patient services. We find actors being guided by and modulating multiple logics, with differing degrees of enthusiasm, integration and awareness. These logics are: ownership responsibility; professionalism; population health managerialism; entrepreneurial commercialism.
