# Supplementary material for: Interacting institutional logics in general dental practice
Source: Soc Sci Med. 2013 Oct;94:63–70. doi: 10.1016/j.socscimed.2013.05.038 (PMC3750214; doi:10.1016/j.socscimed.2013.05.038)
Supplement: Supplementary file 1 [file mmc1.docx]

**Archive data: the evolving of the dental practice organizational field.**

*Between 1980 and 1990*

From the 1980s onwards, professions faced challenges from a political era of New Public Management, with dentistry being no exception. By the end of the decade, the government had instituted an internal market in the NHS, with attention focused on maximising efficiency, quality and consumer responsiveness. Although organisational re-structuring associated with the implementation of an internal market did not affect dental practice directly at the time, the underpinning political philosophy, and environment of budget restraint did have an impact in several ways. In 1985 ‘Promoting Better Health’, a White Paper on primary health care, the government made clear its intention to use dental charges as a way of raising tax revenue. Free dental examinations were abolished. Providers saw this as a ‘breakdown event’, set against a background of improving dental health in the population, where numbers of dentists were out of proportion to need, and competition for patients becoming a concern for dental practitioners.

UK dentistry, along with optical and pharmaceutical services, has had closer ties with the commercial world, than most other aspects of health care delivered under the auspices of the NHS. Contribution of patients to the cost of their dental care has been a feature of NHS dental care almost since the inception of the system. The proportion of care paid by the patient however has grown steadily over the years (although certain groups such as children, pregnant women and those on state benefits remain exempt). Currently non-exempt patients contribute as much as 80% of the cost of their NHS dental care up to a ceiling limit. The government pays the remaining 20% and any additional sum over the ceiling limit, as well as the full cost of care for exempt groups. A growing voice of consumerism during the 1980s; rising patient charges, with media reports portraying dentists as overprescribing, culminated in the 1986 Schanschieff report into unnecessary dental treatment. The dental profession responded by expressing concern that abolishing free dental check-ups indicating government policies subjecting dental practice to quasi-market forces, thus weakening their professional influence on determining the appropriate type and quality of dental services. Government policy created further challenges to dental practice when relaxation of advertising constraints for opticians was followed by similar changes in dentistry. Archival evidence shows mixed responses, with some practices and professional bodies resistant and others cognisant of both the realism and perhaps even opportunity of adopting more entrepreneurial approaches to delivering care.

As well as means of delivery, the nature of what constituted care was also evolving. This period begins with immutable professionally defined standards of care based on expert opinion of need, but ends with cost-calculus finding increasing traction among the profession, the tension being evident throughout the period examined, a spilling into broader concerns with NHS structure. Two individuals, Stephen Noar and Marilyn Orcharton felt any the resolution of delivery of dental care to professionally defined standards, but within the confines of a service delivered at lowest cost, was impossible within the existing structure of the NHS. In 1986 they established *Denplan*, a commercial enterprise able to supplement and challenge the NHS (Watson-James, 1997). Based on a model of patient-funded dental insurance, this new structure (now a major player in the dental practice field), instituted a way in which actors could maintain beliefs and practices based on professionally defined standards of care, whilst using commercial models of delivery. Noar and Orcharton were not alone, the period finding some dental practices willing to tolerate an emergent logic of commercialism, with others still resistant.

*1990-2000*

A new dental contract introducing an element of capitation to a previously exclusively fee-per-item system of remuneration precipitated a haemorrhaging of practitioners from the NHS and a shift to private provision (Lynch and Calnan, 2003). The contract also required dentists to give fuller descriptions of treatment to patients in treatment plans, to use information leaflets, and to guarantee rights of access to emergency care. Although the package was agreed by the BDA, grass roots dentists were unhappy with the settlement (there was a 66% ‘No’ vote to the contract), mainly because of the new funding structure (Gordon, 1991). There were reports of one in five dentists pulling out of the NHS for non-exempt patients, shifting the balance of their practice to private work. This skewing toward private provision became more pronounced in 1992, when, because of unanticipated earnings under a newly instituted capitation arrangement, the Treasury instituted a 7% fee cut (initially proposed as 20%). Expansion of private dentistry, which required marketing and selling of dental services, and a consumer focus, meant many practices becoming overtly commercial; they were private businesses. Encouraged by government legislation strengthening the hand of the consumer, there was increasing recognition that restricting provision to that based purely on clinically identified need may not fit with what patients want and are willing to pay for. The traditional values of care associated with expert providers and passive recipients were breaking down as commercial opportunity became visible.

Following a remuneration review by Kenneth Bloomfield, and an enquiry by the House of Common’s Health Select committee, the government published a response in 1994 outlining alternative options for remuneration that favoured setting up local purchasing systems sensitive to regional differences in needs. A White Paper (Department of Health, 1996) confirmed the government’s intention to pilot a system based on local purchaser/provider arrangements, and a new type of organisational structure was instituted called Personal Dental Services (PDS). A total of 54 schemes involving groups of practices throughout the country were tested between 1998 and 2001. The previous fee-per-item and capitation arrangements had both been administered centrally from a national budget, whereas PDS contract terms and monitoring of performance was undertaken locally and involved local purchasers. PDS pilot arrangements structurally changed the organisational field with the creation of a new central actor – the purchaser – set within quasi-market structures.

*2000-2012*

Inclusion of purchasers was more widely instituted in the organisational field with the passing of the 2003 Health and Social Care Bill. The previously centrally managed scheme of administering the dental budget was replaced with local contracts between practitioners and commissioners employed by Primary Care Trusts (PCTs). Budgets were devolved to a local level and PCT commissioners charged with procuring dental services to meet the needs of local populations. Practices were encouraged to transfer voluntarily from the centrally managed system into local contracts with commissioners, until in 2006 the previous system was abolished, and all remaining practitioners forced to transfer into the new arrangements. 2006 also brought a further change in dental contract currency, with the fee-per-item system replaced with a system based on bands of activity: a new, previously untested arrangement which was widely contested (Harris & Sun, 2012). The 2006 dental contract created a further watershed moment and around 10% of practitioners refused to sign the contract and effectively withdrew completely from providing an NHS service.

Emergence of local commissioning within the organisational field meant that when a dental practice exited to the private sector, the contract could be re-commissioned to another NHS provider, usually via a tendering process. This created new market opportunities for actors to expand practices or set up new practices in different areas. Government removal of restrictions on the number of Bodies Corporate (DBCs) in 2006 made market entry easier for organizations using commercialized models of delivering dentistry. The organizational field became increasingly diverse. There are several large chains of dental practices (DBCs), and many private practices providing dentistry according to a commercial model; as well as a range of NHS practices with differing levels of commitment to NHS work, along with specialist legal/accounting practices, technological advances in equipment and treatment supply, emerging markets in hygiene and cosmetic treatment (Willcocks, 2012). By 2010 NHS dental service availability returned to pre-2006 levels and the government, recognising shortcomings in the 2006 contract and following an Independent Review (Department of Health, 2009), agreed to pilot a ‘new’ dental contract (due to be implemented in 2014).

**Table 1 Timeline of Key Events in the English Dental Practice System**

| **Date** | **Event** |
| --- | --- |
| 1948 | NHS is established following passage of the National Health Service Act 1946, and general dental practice care is made available to everyone, free at the point of delivery, provided they can find a dentist |
| 1951 | Legislation introducing patient charges for dentures |
| 1981 | Government Dental Strategy Review Group reports |
| 1985 | Patient charges for dental examinations introduced |
|  | General Dental Council amends regulation on advertising |
| 1986 | Schanschieff Committee of Enquiry into Unnecessary Dental Treatment |
| 1987 | Government White paper ‘Promoting Better Health’ |
| 1990 | New dental contract introduces registration, written treatment plans, practice leaflets |
| 1992 | Fee Cut and Bloomfield Report |
| 1993 | House of Commons Health Select Committee on Dental Services |
| 1996 | Government White paper ‘Choice and Opportunity – primary care: the future’ |
| 1998 | Personal Dental Services (PDS) schemes established |
| 2001 | House of Commons Health Select Committee on Access to NHS Dentistry |
| 2002 | NHS Dentistry: Options for Change vision for NHS dentistry |
| 2003 | Health and Social Care Act 2003 Primary Care Trusts (PCTs) to be responsible (from April 2005) for contracting locally with dentists as part of their commissioning role |
| 2006 | New dental contract based on Bands courses of treatment with new banded system of patient charges |
|  | Removal of restrictions on number of Dental Bodies Corporate |
| 2008 | General Dental Council previously comprised of members elected by the profession replaced by independently appointed professionals and lay members |
|  | House of Commons Health Select committee |
| 2009 | Steele Independent Review of NHS Dental services |
| 2012 | New dental contract piloted |
| 2013 | PCTs abolished and commissioning role taken by NHS Commissioning Board |

**References**

Department of Health. (1996). *Choice and Opportunity – primary care: the future*. London: HMSO.

Department of Health (2009). *NHS dental services in England. An independent review* *led by Professor Jimmy Steele*. London: Department of Health.

Gordon, E. (1991). *Dentists – the crunch still to come*. British Dental Association News. London: BDA.

Harris, R., & Sun, N. (2012). Translation of remuneration arrangements into incentives to delegate to English dental therapists. *Health Policy*, *104*, 253-259.

Lynch, M., & Calnan, M. (2009). The changing public/private mix in dentistry in the UK- a supply side perspective. *Health Economics* *12*, 309-321.

Watson-James, D. (1997). The shape of things to come? *British Dental Journal* *182*, 31-34.

Welsh valley practitioner. (1985). Letter: Has the profession lost its way? *British Dental Journal,* *15*,173.

Willcocks, S. (2012). The entrepreneurial role in primary care dentistry. *British Dental Journal*, *212*, 213-217.
